# Neuroprotective Potential of Pyranocoumarins from *Angelica gigas* Nakai on Glutamate-Induced Hippocampal Cell Death

**DOI:** 10.3390/antiox12081651

**Published:** 2023-08-21

**Authors:** Nguyen Khoi Song Tran, Tuy An Trinh, Jaesung Pyo, Chang Geon Kim, Jae Gyu Park, Ki Sung Kang

**Affiliations:** 1 College of Korean Medicine, Gachon University, Seongnam 13120, Republic of Korea; kauri87@gachon.ac.kr; 2 Saigon Pharmaceutical Science and Technology Center, University of Medicine and Pharmacy at Ho Chi Minh City, Ho Chi Minh 70000, Vietnam; ttan@ump.edu.vn; 3 College of Pharmacy, Kyungsung University, Busan 48434, Republic of Korea; jspyo@ks.ac.kr; 4 Advanced Bio Convergence Center (ABCC), Pohang Technopark Foundation, Pohang 37668, Republic of Korea; rjs6538@ptp.or.kr

**Keywords:** *Angelica gigas*, decursin, glutamate, HT22 cell line, neuroprotection

## Abstract

Chronic neurodegenerative diseases are typically associated with oxidative stress conditions leading to neuronal cell death. We aimed to investigate the neuroprotective effect of three pyranocoumarins (decursin, decursinol angelate, and decursinol) targeting oxidative stress factors. Decursin (also known as dehydro-8-prenylnaringenin) is a prenylated coumarin compound consisting of a coumarin ring system with a prenyl group attached to one of the carbons in the ring. As a secondary metabolite of plants, pyranocoumarin decursin from *Angelica gigas* Nakai presented protective effects against glutamate-induced oxidative stress in HT22, a murine hippocampal neuronal cell line. Decursinol (DOH) is a metabolite of decursin, sharing same coumarin ring system but a slightly different chemical structure with the prenyl group replaced by a hydroxyl group (-OH). In our findings, DOH was ineffective while decursin was, suggesting that this prenyl structure may be important for compound absorption and neuroprotection. By diminishing the accumulation of intracellular reactive oxygen species as well as stimulating the expression of HO-1, decursin triggers the self-protection system in neuronal cells. Additionally, decursin also revealed an anti-apoptotic effect by inhibiting chromatin condensation and reducing the forming of annexin-V-positive cells.

## 1. Introduction

Neurodegeneration-related diseases are defined by the dysfunction or loss of neurons in the central nervous system resulting in dementia or ataxia. Therein, the vulnerable adult hippocampal neurogenesis (AHN) were reported as elevated factor of neuronal dysfunction in age-related diseases [1]. Losing their memory and social cognition can put the patients’ life at risk [2]. The popular neurodegenerative disorders that have been studied are Alzheimer’s disease (AD), Parkinson’s disease (PD), multiple sclerosis (MS), and amyotrophic lateral sclerosis (ALS) [3]. Among them, Alzheimer’s disease represents 60–70% of diagnosed dementia cases in elderly populations worldwide [4]. Since neurodegenerative diseases appear to have no current available treatment, early diagnosis and optimizing physical health and abnormal psychological symptoms are the goals of neurological disorder therapies.

Besides genetic factors, most neurodegenerative diseases are consequences of neuronal toxicity or oxidative stress. The accumulation of products of aerobic respiration called reactive oxygen species (ROS) causes lipid peroxidation, following by cell membrane and lipoprotein damage. Therefore, endogenous ROS scavengers play a very important role in neuroprotection. Some of the endogenous antioxidant enzymes that are directly regulated by the nuclear factor Nrf2 signaling pathway are heme oxygenase-1 (HO-1) and glutathione (GST) [5]. Oxidative stress leads to the translocation of Nrf2 from the cytosol to the nucleus, activating the antioxidant response element (ARE) to trigger the transcription of cytoprotective genes including HO-1 [6]. Heat shock proteins (HSPs) and immediate early genes (IEGs) are the stress proteins induced in response to cellular stress. HSPs are involved in chaperone functions that prevent incorrect protein folding and aggregation [7]. HSP32, together with HSP27 and HSP70, are the three main HSPs that respond to brain injuries [8]. Heme oxygenase-1 (HO-1; also known as HSP32) is another heat shock protein which takes part in the inflammatory mediation consisting of neuroinflammation in the central nervous system. Oxidative stress with an excess of ROS is one of the common factors that induces the HO-1 response [9].

Moreover, when xenobiotics such as foods, medicines or chemicals are unexpectedly present in individuals’ bodies, they will become targets of endogenous enzyme metabolism as a protective response. This response is carried out by three groups of enzymes which are known as drug metabolizing enzymes (DMEs). While phase I group is in charge of xenobiotic oxidization and hydrolyzation, phase II enzymes conjugate or degrade the metabolites, and phase III is responsible for the excretion of these metabolites [10]. Hence, phase II of drug metabolism plays a key role in the whole endogenous protective procedure [11]. Therein, the Nrf2/ARE pathway has been proven to be a major regulator of the phase II group which can be induced by products of phase I [12]. Nrf2 activities in neurons were reported to emerged with certain enzymes in phase II consisting of detoxifying enzyme HO-1 and NAD(P)H: quinone oxidoreductase (NQO1) acting as neuroprotective antioxidants [13]. These mechanisms protect physical health from acute neurological and neurodegenerative diseases.

Natural compounds from medicinal plants are considered potential candidates for the prevention and supportive treatment of neurodegenerative diseases [14]. The neuroprotective effects of natural compounds have been demonstrated in various mechanisms including free radical scavenging activities and anti-apoptotic/necrosis signaling pathways. Many phytochemicals presented neuroprotective effects in previous research such as curcumin [15], kaempferol [16], quercetin, epigallocatechin gallate [3], polygeline [17], and gartanin [18]. *Angelica gigas* Nakai belonging to Apiaceae family is a biennial plant and classified as a short-lived perennial plant. The root of *A. gigas* was used in oriental medicine for the treatment of anemia, migraine headaches, arthritis, injuries, and gynecological diseases [19]. The main bioactive components of *A. gigas* roots that have been studied are coumarins, polyphenols, phthalides, essential oils, and polysaccharides [20]. Therein, the pyranocoumarins from *A. gigas* have been found to have many pharmacological properties like anti-inflammatory [21,22], anticancer [20,23,24,25], and neuroprotective properties [26,27,28]. Pyranocoumarins also act as free radical scavengers in human blood [29,30].

Previous work from Lee et al. proved the neuroprotective effect of the root of *Angelica gigas* Nakai (Apiaceae) on a mouse stroke model [31]. In the present study, we aimed to investigate this effect of three pyranocoumarins (decursin, decursinol angelate (DA), and decursinol (DOH)) which were prepared from the roots of *A. gigas* (as described in the materials and methods) on glutamate-induced apoptosis in HT22 murine hippocampal neuronal cells. Although playing a critical role in brain functions as an excitatory neurotransmitter of the central nervous system, an overbalance of glutamate release induces excitotoxicity and oxidative stress, which leads to neuronal cell death [32]. Programmed cell death, apoptosis, is characterized by changing chromatin from a heterogeneous active form to an inert, highly condensed form and genomic DNA fragmentation. Together with cell membrane blebbing, these hallmarks of the terminal stages were applied for apoptosis research [33]. The intracellular ROS level, chromatin condensation, and apoptotic annexin V-positive cells were evaluated to check the efficacy of *A. gigas* Nakai-extracted compounds in inhibiting apoptotic neuronal cell death. Biomarker expression levels were also assessed to clarify the underlying protective mechanism of these bioactive compounds on glutamate-induced neurotoxicity.

## 2. Materials and Methods

### 2.1. Materials

The experimental reagents and HPLC-grade solvents were obtained from Sigma Aldrich (St. Louis, MO, USA). The *Angelica gigas* Nakai was purchased from Hamyang-gun, Gyeongsangnam-do.

### 2.2. Extraction of Angelica gigas Nakai

Ground Angelica (100 g) was extracted for 24 h with 0.3 L of 95% ethanol at room temperature and filtered. The extract was incubated at −20 °C for 10 h and then centrifuged at 5000 rpm for 10 min, and then ethanol was evaporated. To obtain a final decursin (JP-203) and DA (JP-204) mixture of 3 g, the residue was vortexed with 60% ethanol of 0.5 L and eluted [34].

### 2.3. Separation of Decursin (JP-203) and DA (JP-204)

The extracted decursin (JP-203) and DA (JP-204) mixture was separated by recycling HPLC. The column used was a JAIGEL ODS-AP (20 × 500 mm). A 0.01% formic acid in 70% acetonitrile solution was used as the mobile phase. The oven temperature was set at 30 °C. The flow rate was 5 mL/min. The wavelength of the UV detector was 329 nm [34]. The JP-203 and JP-204 mixtures were made at a 15–17% concentration.

### 2.4. The Conversion of Decursin (JP-203) and DA (JP-204) to DOH (JP-202)

Lithium hydroxide was added to the mixture of decursin (JP-203) and DA (JP-204) in a tetrahydrofuran: H_2_O (4:1) solution while stirring at room temperature. Tetrahydrofuran was removed by decompression. The pH of the aqueous layer was adjusted to pH 4 and then extracted with ethyl acetate. The residue of the solvent after evaporation in vacuo was purified in silica gel with flash column chromatography to obtain DOH (JP-202) (12–16%) [35].

### 2.5. HPLC Analysis

The employed HPLC equipment was a Thermo Scientific UltiMate 3000 HPLC with Chromeleon 7. Phages separations were performed using an Agilent C18 column (4.6 × 250 mm, 5 μM). The flow rate was adjusted to 1.0 mL/min. Also, the detection wavelength was 330 nm. Water (solvent A) and 95% acetonitrile (solvent B) were used as the mobile phase. The gradient elution protocol was as follows: 0–0.1 min, 80:20; 0.1–15 min, 10:90; 15–16 min, 80:20; 16–18 min, 80:20 [36].

### 2.6. Cell Culture

The HT22 murine hippocampal neuronal cells were acquired from ATCC and cultured in Dulbecco’s Modified Eagle Medium (Corning, Manassas, VA, USA) supplemented with 10% fetal bovine serum (Atlas Biologicals, Fort Collins, CO, USA) and 100 units/mL penicillin and 100 mg/mL streptomycin (P/S) (Gibco, Grand Island, NY, USA). Cells were maintained in an incubator with a 37 °C humidified atmosphere and 5% CO_2_. The cell confluency was evaluated every two days.

### 2.7. Cell Viability Assay

HT22 cells were grown in 96-well plates at a density of 1 × 10^4^ cells/well for 24 h. Then, the cells were treated with test samples (JP-202, JP-203, and JP-204) at varying concentrations (3.125, 6.25, 12.5, 25, and 50 μM) to evaluate their protective effect against cell death induced by 5 mM glutamate. After 24 h of treatment, to determine cell viability, 10 μL EZ-Cytox assay reagent (DoGen, Seoul, Republic of Korea) was added to every single well and incubated for 1–4 h. The colorimetric absorbance was measured at 450 nm with a microplate reader (PowerWave XS; Bio-Tek Instruments, Winooski, VT, USA).

### 2.8. Hoechst 33342 Staining

A density 2 × 10^5^ cells/well of HT22 cells were seeded into 6-well plates for 24 h. After that, the cells were exposed to 25 μM of JP-203 for 12 h in the presence or absence of 5 mM glutamate. Then, the cells’ chromatin was marked with Hoechst 33342 and chromatin condensation was observed by fluorescence microscopy.

### 2.9. ROS Assay

HT22 cells were cultivated in 96-well plates the day before and then treated with 5 mM glutamate and JP-203 at 25 and 50 μM for 8 h. After that, the cells were stained with 10 M 2′,7′-dichlorodihydrofluorescein diacetate (H2DCF-DA) to detect the accumulation of ROS. The fluorescence intensities were measured by a fluorescence microplate reader, with 488 nm excitation and 525 nm emission fluorescent peaks.

### 2.10. Western Blotting Analysis

HT22 cells were seeded into 6-well plates at 2 × 10^5^ cells/well for 24 h and then treated with JP-203 at 25 and 50 μM in the presence or absence of 5 mM glutamate. After 6 h, cell pellets were collected and washed with DPBS. RIPA buffer supplemented with protease inhibitor cocktail (1X) was added to obtain whole-cell protein extracts according to the manufacturer’s instructions. The protein concentration of the cell extracts was evaluated using the Pierce™ BCA Protein Assay Kit (Thermo Scientific, Waltham, MA, USA).

Each 10 μg of extracted samples was separated by SDS-PAGE and blotted onto PVDF transfer membranes. The primary antibodies included HO-1 and glyceraldehyde 3-phosphate dehydrogenase (GAPDH) (Cell signaling Technology, Danvers, MA, USA) and horseradish peroxidase (HRP)-conjugated secondary antibodies (Cell Signaling, USA) were used to label the target proteins. The blots were developed using PierceTM ECL Advance Western Blotting Detection Reagents (Thermo Scientific, Waltham, MA, USA) and detected with FUSION Solo Chemiluminescence System (PEQLAB Biotechnologie GmbH, Erlangen, Germany).

### 2.11. TALI Assay

The hippocampal HT22 cells were plated in 6-well plates at a population of 2 × 10^5^ cells/well for 24 h. Next, the cells were treated with 5 mM glutamate and JP-203 at the indicated concentration (25 and 50 μM). After 12 h, cell pellets were collected and washed in DPBS. The cells were later stained with annexin V–Alexa Fluor 488 to measure apoptosis and propidium iodide to count the number of dead cells.

### 2.12. Statistical Analysis

The bar graphs show the mean ± standard deviation (SD). Statistical significance was determined using Student’s *t*-tests. *p* ≤ 0.05 was considered statistically significant.

## 3. Results

### 3.1. Decursin (JP-203) and Its Derivatives Isolated from A. gigas Protected HT22 Cell from Glutamate-Induced Cell Death

**Figure 1:**
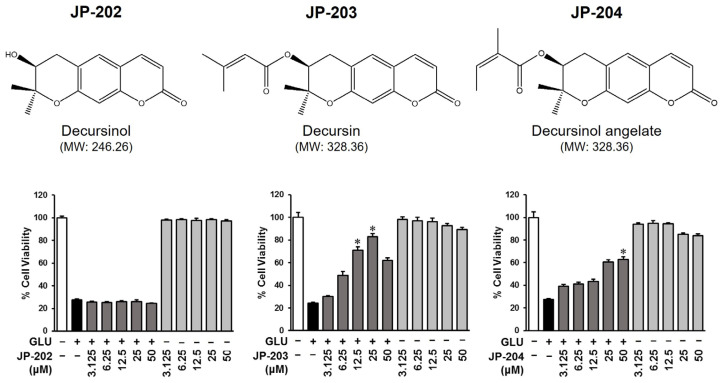
Protective effects of pyranocoumarins extracted from A. gigas on HT22 cell death induced by glutamate. HT22 cells were cultured in 96-well plates at a density of 1 × 10^4^ cells/well. After 24 h, cells were treated with test samples at a range of concentrations (3.125–50 μM) to evaluate their protective effects against cell death induced by 5 mM glutamate. The EZ-Cytox assay was employed to determine cell viability 24 h after the treatment. (*) *p* < 0.05 vs. glutamate-treated group indicates significant difference.

The neuroprotective effect of pyranocoumarins and its derivatives from *A. gigas* roots were first evaluated through cell cytotoxicity assays measuring glutamate-induced murine hippocampal neuronal HT22 cell death. The cells were cultured for 24 h and exposed to glutamate, decursinol (JP-202), decursin (JP-203), and decursinol angelate (JP-204) compounds. While glutamate treatment induced neuronal apoptosis of HT22 cells, treatment with the other compounds appeared to have no effect on cell viability. Moreover, the JP-203 and JP-204 co-treated with glutamate groups presented a significant recovery from apoptotic induction. As shown by the results, the exposure of HT22 to 5 mM glutamate diminished the number of living cells to 24.26 ± 0.73% while treatment with pyranocoumarin compounds recovered the cell numbers in a dose-dependent manner (Figure 1).

Of the different treatments, treatment with JP-203 at concentrations of 12.5 and 25 μM significantly improved the cell viability to 70.78 ± 3.27% and 82.95 ± 2.81%, respectively, compared to the induced group. Moreover, JP-204, an isomer of JP-203, also rescued the cells at a dose of 50 μM. However, JP-202 showed no effect on HT22 cell viability with or without apoptosis induction.

### 3.2. Decursin (JP-203) Prevented the Oxidative Stress-Induced Chromatin Condensation and Neuroinflammation in HT22 Cells Treated with Glutamate

**Figure 2:**
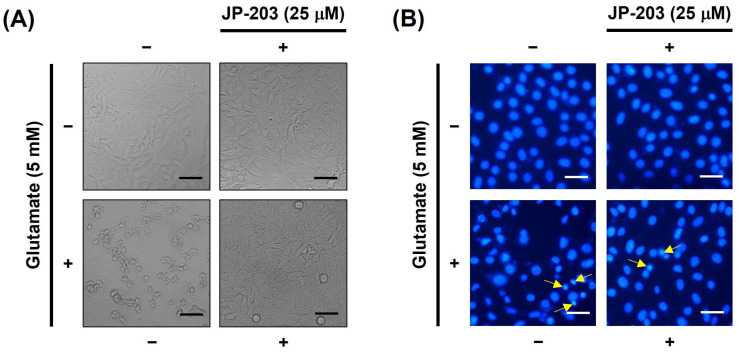
JP-203 prevented the chromatin condensation in HT22 cells treated with glutamate. (**A**) Bright field microscope image of morphologies of HT22 cells treated with 5 mM glutamate and 25 μM JP-203 for 24 h. (**B**) HT22 cells after treatment with glutamate and JP-203 at a dose of 25 μM for 12 h were stained with Hoechst 33342 and the chromatin condensation was observed by fluorescence microscopy. The yellow arrow indicates condensed chromatin. Scale bar indicates 20 μm.

To examine the protective effect of decursin on HT22 neural cells, we conducted four groups of in vitro tests including non-treatment, glutamate-induced apoptosis, JP-203 only, and glutamate and JP-203 co-treatment groups. The morphology examination in bright field images (Figure 2A) revealed that cell death condition after glutamate exposure was recovered in the JP-203 co-treated group. The Hoechst 33342 staining was applied for condensed chromatin determination. According to the fluorescence microscopic images (Figure 2B), treatment with decursin (JP-203) at a concentration of 25 μM clearly attenuated the chromatin condensation of glutamate-induced HT22 cell death. The amount of condensed DNA (yellow arrow) with brighter and smaller shapes than normal nuclear DNA, was lower in the JP-203 co-treated group in comparison with the control group.

**Figure 3:**
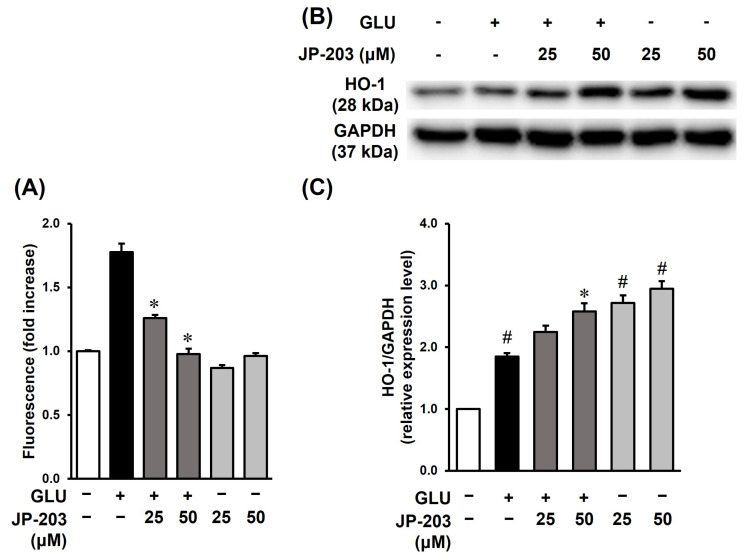
The protective effect of JP-203 on oxidative stress resulting from excessive glutamate in HT22 cells. (**A**) JP-203 diminished the accumulation of intracellular ROS in HT22 cells which was stimulated by glutamate. HT22 cells were incubated with 5 mM glutamate and JP-203 compound at concentrations of 25 and 50 μM. After 8 h, cells were stained with 10 μM 2′,7′-dichlorodihydrofluorescein diacetate (H2DCF-DA) to detect the accumulation of ROS. (**B**) JP-203 stimulated the expression of HO-1. Cells were treated with JP-203 at the indicated concentrations in the presence or absence of glutamate for 6 h and then total protein was collected for Western blotting analysis. The target proteins were detected by conjugation with epitope-specific primary and secondary antibodies. (**C**) The quantitative graph of relative expression levels of HO-1 to GAPDH from the Western blot analysis. (*) *p* < 0.05 vs. glutamate-treated group; (#) *p* < 0.05 vs. non-treated group.

Oxidative stress triggers neuronal cell death through both the necrotic and apoptotic signaling pathways. Excessive glutamate in the central nervous system leads to oxidative stress which is characterized by the accumulation of intracellular free radicals [37]. We performed ROS assays with four experimental groups including non-treatment, glutamate, JP-203, and glutamate and JP-203 co-treated groups. The ROS level was investigated by fluorescent intensity of H2DCF-DA. As a result, treatment with decursin successfully diminished oxidative stress through a reduction in ROS accumulation, which was sharply induced by glutamate, to normal levels (Figure 3A).

Moreover, Western blot analysis was performed to clarify the underlying mechanism of the reaction between JP-203 and the oxidative stress-response enzymes. As displayed in Figure 3B, with or without glutamate stimulation, the presence of JP-203 elevated the protein level of heme oxygenase-1 (HO-1), an inducible enzyme that takes part in the mediation of inflammation, consisting of neuroinflammation in the central nervous system. Additionally, HO-1 also plays an important role in anti-apoptotic enzyme responses and atherosclerotic disease [38,39].

Oxidative stress with a surplus of ROS is one of the common factors that induces the HO-1 response [9]. It is known that HO-1 transcription is regulated by the nuclear factor erythroid 2-related factor 2 (Nrf2). In general, oxidative stress leads to the translocation of Nrf2 from the cytosol to the nucleus, activating the antioxidant response element (ARE) and triggering the transcription of cyto-protective genes including HO-1 [6]. HT22 cells were exposed to glutamate-induced neurotoxicity and decursin at concentrations of 25 and 50 µM for 6 h. Western blot analysis showed that the expression of HO-1 protein in HT22 cells was stimulated in a decursin-concentration-dependent manner (Figure 3B). The dose-dependent elevation of HO-1 with or without glutamate stimulation is presented as quantitative results (Figure 3C) which also correlated with the blotting images.

### 3.3. Decursin (JP-203) Ameliorates Apoptosis Index of Glutamate-Induced HT22 Cell Death

**Figure 4:**
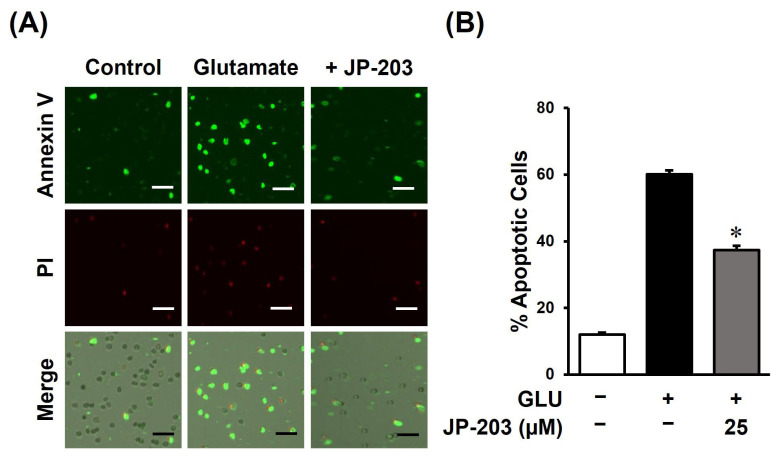
The anti-apoptotic activity of JP-203 against glutamate-induced HT22 cell death. (**A**) The microscopy pictures from TALI image-based cytometric analysis of JP-203. After treatment with 5 mM glutamate and 25 µM JP-203 for 12 h, cells were stained with annexin V–Alexa Fluor 488 for apoptotic cell identification or propidium iodide (PI) for dead cell labeling. (**B**) The comparative graph illustrating the percentage of apoptotic cells between the JP-203 treated and untreated groups was made using counting of annexin-V-stained cells. (*) indicates *p* < 0.05 vs. glutamate-treated group. Scale bar indicates 20 μm.

The common signs of apoptosis are cell shrinkage, nuclear fragmentation, plasma membrane blebbing, and chromatin condensation [40]. As mentioned before, decursin inhibited the chromatin condensation in the progress of apoptotic cell death caused by glutamate-induced oxidative stress. Therefore, we conducted a TALI assay with control, glutamate, and co-treatment of glutamate and JP-203 groups to verify the anti-apoptotic effect of decursin. The apoptotic cells were labeled with annexin V–Alexa Fluor 488 dye and the dead cells were characterized by propidium iodide staining. In Figure 4A, the fluorescent microscopy images demonstrated a reduction in annexin-V-as well as PI-stained cells in the JP-203 co-treated group indicating a rescue of glutamate-induced cytotoxicity by decursin. Therefore, the comparative graph also revealed that glutamate significantly stimulated the apoptotic cell death to 60 ± 1.20% whereas treatment with decursin at a concentration of 25 µM diminished it to 37 ± 1.33% (Figure 4B).

## 4. Discussion

Recent studies exhibited that *A. gigas* Nakai appears to have neuroprotective effects in mouse and rat models. A methanol extract of *A. gigas* Nakai improved the abnormal morphology and infarction volume of tMCAO mouse brains (a stroke model) by attenuating ERK-related MAPK signaling pathways [31]. The root and single compound extracts of this herbal remedy have been recognized as neuroprotective and cognitive enhancers in animal models as well [28]. Moreover, an experimental report on rat ischemic brains established the protective actions of *A. gigas* Nakai extracts including stabilizing the blood–brain barrier (BBB) permeability and inhibiting the elevation of astrocyte numbers [41]. As mentioned previously, decursin is a major bioactive component of *A. gigas* Nakai and could play a certain role in its neural protection mechanisms.

Our study revealed that decursin, a prenylated coumarin compound consisting of a coumarin ring system with a prenyl group, improved the cell viability induced by glutamate in HT22 cells at doses of 12.5 and 25 μM (Figure 1). Moreover, DA, an isomer of decursin, also recovered the apoptosis condition to 65% at a dose of 50 μM. However, DOH, a metabolite of decursin with a simple hydroxyl group instead of a prenyl group, showed no rescue effect on HT22 cells. This could be due to the permeability of cell membranes to certain compounds. Since mammalian cells are constructed with a double-layer lipid membrane, hydrophobic molecules which have a high affinity with lipids will easily be transferred to intracellular cytostomes via passive diffusion. The post-translationally modified prenylation increases the lipophilicity of natural products which means that decursin has a higher diffusion ability to the cell membrane than the other two modified compounds [42].

This higher absorbability of decursin by the cell membrane was also observed in the previous report from Zhang et al. about the distribution of decursin, DA, and DOH in rodent and human bodies [43]. When an individual takes a medication, the drugs interact with the body reacting system prior to being delivered to target tissues. Studies on these actions are called pharmacokinetic studies. Pharmacokinetic parameters include the absorption, distribution, metabolism, and elimination of certain medicines. The maximum concentration of a drug in the plasma and the drug’s volume of distribution are primary pharmacokinetic parameters in evaluating its therapeutic efficiency. In brief, the distribution of a drug in the absence of elimination is an inverted proportion of plasma maximum concentration.

Cmax = D/Vd

where Cmax is the plasma concentration of the drug, D is the total amount of drug administered, and Vd is the distribution of the drug in ideal conditions. Zhang et al. proved that the plasma level of DOH is roughly 400 and 700 times higher than decursin in human and rodent plasma, respectively. This means that the tissue/plasma ratio of decursin is much greater than that of DOH in both models. The study also found that the termination half-life t1/2 of decursin and DA were much longer than that of DOH (17.4 and 19.3 vs. 7.4 h) (Table 1). Therefore, the exposure time of decursin and DA to the human body was significantly increased compared to that of DOH.

There are many pathogenic factors leading to injury of the brain and central nervous system. Since neurodegeneration can be the result of genetic and environmental issues, the research directions and therapeutic approaches are varied as well. Among the various external factors, chronic exposure of neural cells to certain neurotransmitter chemicals and metals causes toxicity to neurons. The work of Li et al. demonstrated the protective effect of decursin isolated from *A. gigas* Nakai on amyloid-25-35-induced apoptosis of adrenal pheochromocytoma PC12 cells by suppressing mitochondria-related caspase expression [44]. This group also found elevated nuclear transcription factor Nrf2 regulation of antioxidant enzymes with decursin treatment [26]. In addition, decursin and DOH have been shown to improve cortical cell death caused by excessive glutamate [45]. Previous data also indicated that abnormal glutamatergic neurotransmission systems altered the hippocampus of Alzheimer patients via N-methyl-D-aspartate (NMDA) receptors [46,47]. We focused on the rescue effect of JP-203 on cell toxicity of surplus glutamate on the murine hippocampal neuron HT22 cell line through the inhibition of oxidative stress. An imbalance in ROS formation during neurotoxicity followed by excess production of certain proteins, including β-amyloid and α-synuclein, can result in major neurodegenerative disorders [16]. In the present study, treatment of HT22 cells with glutamine resulted in oxidative stress and apoptosis, in which the overabundance of ROS induced the HO-1 response and chromatin condensation. This finding demonstrated the protective ability of decursin (JP-203) against oxidative stress-induced apoptosis in cells (Figure 3). The change in HO-1 protein expression might be regulated by certain signaling pathways that should be investigated further. Consistent with our results, a study by Song et al. investigated the antioxidant-like activity of decursin via AMPK pathway activation in AA+-induced apoptosis of human HepG2 cells and a murine liver injury model [48].

Excess glutamate, an excitatory neurotransmitter controlling synaptic signals in the mammalian brain, leads to ROS-dependent neuronal cell death. Many hallmarks are involved in the subcellular shifts during apoptosis, and the condensation of nucleus chromatin is one of the obvious signs. Chromatin impaction has been shown to be an indicator during apoptotic process in brain dysfunction [49]. From Hoechst 33342 staining fluorescence microscopy images, decursin treatment at 25 μM also prevented the chromatin condensation in glutamate induced HT22 cell death (Figure 2). Therefore, decursin might play a key protective role against DNA fragmentation and inert chromatin condensation in apoptotic neuronal cells. Additionally, major changes in neuronal cell morphology can be detected when cells undergo the apoptotic process. The TALI assay showed a remarkable reduction of annexin V–Alexa Fluor 488-labeled cells in the glutamate-induced HT22 group co-treated with decursin (Figure 4). Not only does it protect neurons from programed cell death, decursin also has the ability to cross through blood vessels. The previous finding of Madgula et al. using the TEER values of Caco-2 and MDR-MDCK cell mono layers suggested that decursin can potentially transport across the BBB [50]. Another study on brain ischemic injury reflected a protective action of decursin on pyramidal neurons of the hippocampus in gerbil forebrains [51]. This study suggested a BBB-crossing capacity of decursin through protecting against the astrocyte endfeet damage caused by brain ischemia in a rodent animal model.

In conclusion, decursin exerted a significant protective effect against murine hippocampal HT22 neuronal cell death induced by glutamate among the pyranocoumarins isolated from *A. gigas*. Our findings proved that decursin reduced the accumulation of ROS in cellular oxidative stress conditions. By activating the expression of the HO-1 enzyme, decursin triggered the antioxidant self-defense system of HT22 cells in response to excess exogenous glutamate. Decursin also prevented apoptotic neuronal cell death, one of the consequences of oxidative stress.

## Figures and Tables

**Table 1:** The primary pharmacokinetic parameters of decursin, DA, and DOH in human models (n = 20) versus those in rats. Reprinted with permission from [43]). Copyright (2015) Plos One publications.

Analyte → PK Parameter	D	DA	DOH	Statistical Analyses	Linear Coefficient of Determination
**Human**				*p* value (post-hoc power)	r^2^ values
***T_max_*, h, Mean (SD)**	2.1 (1.2)	2.4 (1.4)	3.3 (1.6)	Paired *t*-test, 1-sided	D vs. age r^2^ = 0.035
				D vs. DA *p* = 0.0094 (20%)	D vs. weight r^2^ = 0.0201
				D vs. DOH *p* = 0.0002 (80%)	DA vs. age r^2^ = 0.0014
				DA vs. DOH *p* = 0.0023 (51%)	DA vs. weight r^2^ = 0.0261
					DOH vs. age r^2^ = 0.096
					DOH vs. weight r^2^ = 0.071
***C*_max_, nmol/L Mean (SD)**	5.3 (4.7)	48.1 (56.4)	2480.3 (842.2)	Paired *t*-test, 1-sided	D vs. age r^2^ = 0.0492
				D vs. DA *p* = 0.0010 (92%)	D vs. weight r^2^ = 0.0214
				D vs. DOH *p* < 0.0001 (>95%)	DA vs. age r^2^ = 0.0053
				DA vs. DOH *p* < 0.0001 (>95%)	DA vs. weight r^2^ = 0.024
					DOH vs. age r^2^ = 0.0001
					DOH vs. weight r^2^ = 0.0004
**AUC_0–48h,_ nmol/L, Mean (SD)**	37.1 (29.2)	335.4 (398.0)	27,579 (13,769)	Paired *t*-test, 1-sided	D vs. age r^2^ = 0.164
				D vs. DA *p* = 0.0011 (92%)	D vs. weight r^2^ = 0.2178
				D vs. DOH *p* < 0.0001 (>95%)	DA vs. age r^2^ = 0.009
				DA vs. DOH *p* < 0.0001 (>95%)	DA vs. weight r^2^ = 0.059
					DOH vs. age r^2^ = 0.129
					DOH vs. weight r^2^ = 0.005
Terminal *t*_1/2_, h, Mean (SD) *	17.4 (6.8)	19.3 (8.5)	7.4 (2.0)	Paired *t*-test, 1-sided	D vs. age r^2^ = 0.0106
				D vs. DA *p* = 0.2406	D vs. weight r^2^ = 0.0186
				D vs. DOH *p* < 0.0001 (95%)	DA vs. age r^2^ < 0.0001
				DA vs. DOH *p* < 0.0001 (>95%)	DA vs. weight r^2^ = 0.0043
					DOH vs. age r^2^ = 0.2927
					DOH vs. weight r^2^ = 0.001
**Rat (n = 3)**					
***T*_max_, h, median (range)**	1 (0.5–2)	1 (0.5–2)	4 (3–8)		
***C*_max_, nmol/, Mean (SD)**	7.3 (4.0)	7.3 (3.4)	5638 (378)		
**AUC_0–48h_, h nmol/L, Mean (SD)**	36.0 (14.3)	64.3 (8.8)	81,272 (6829)		

* The excluded terminal half-life of outliers.

## Data Availability

The data presented in this study are available on request from the corresponding author.
